# Effect of intestinal microbiota transplantation on chronic hepatitis B virus infection associated liver disease

**DOI:** 10.3389/fmicb.2024.1458754

**Published:** 2024-09-11

**Authors:** Lisi Deng, Xiaozhen Guo, Jiehua Chen, Baoyi Li, Na Liu, Jinyu Xia, Mengdang Ou, Zhongsi Hong

**Affiliations:** Department of Infectious Diseases, the Fifth Affiliated Hospital, Sun Yat-sen University, Zhuhai, China

**Keywords:** chronic hepatitis B virus infection, microbiome, metabolome, intestinal microbiota transplantation, alterations

## Abstract

**Background:**

Research on the effects of intestinal microbiota transplantation (IMT) on chronic HBV infection (CHB) progression associated liver disease (HBV-CLD) and alterations in the microbiota post-IMT are quite limited for the moment.

**Methods:**

By integrating microbiome with metabolome analyses, we aimed to the function of IMT and the alterations of gut microbiota in patients with HBV-CLD. First, this study included 20 patients with HBV-CLD and ten healthy controls. Then, 16 patients with CHB were given IMT with donor feces (heterologous) via oral capsule. Fecal samples from CHB patients were obtained before and after IMT, as well as healthy controls, for 16S rDNA sequencing and untargeted metabolomics analysis.

**Results:**

The proalbuminemia were significantly increased after IMT, and the HBsAg and TBA showed a significant decrease after IMT in the HBV-CLD patients. There was statistical difference in the Chaol indexes between between CHB patients and healthy controls, suggesting a lower abundance of the gut microbiota in HBV-CLD patients. In addition, there was statistical difference in the Shannon and Simpson indexes between prior to IMT and post-IMT, indicating that the impaired abundance of the gut microbiota had been improved after IMT. The host-microbiota-metabolite interplay, amino acid metabolism, nicotinate and nicotinamide metabolism, starch and sucrose metabolism, steroid biosynthesis, and vitamins metabolism, were significantly lower in HBV-CLD patients than healthy controls.

**Conclusion:**

IMT may improve the therapeutic effects on patients HBV-CLD. Furthermore, IMT appears to improve amino acid metabolism by impaired abundance of the gut microbiota and therefore improve liver prealbumin synthesis.

## Introduction

Hepatitis B virus infection-associated liver disease (HBV-CLD) places a significant economic burden on the state and society, despite ongoing good interventions in many countries and areas. In the normal course of HBV infection, an estimated 15–40% of individuals with chronic hepatitis B (CHB) infection develop to cirrhosis, liver failure, or hepatocellular carcinoma (Arora et al., 2018).

The ideal endpoint for patients with HBV (both HBeAg positive and HBeAg negative) infection is hepatitis B surface antigen (HBsAg) loss, with or without seroconversion to anti-HBs. This is associated with complete and definitive remission of the activity of HBV (Chotiyaputta and Lok, 2010; Chen et al., 2002). During the natural history of chronic HBV infection, the loss of serum HBeAg and the development of anti-HBe antibodies (HBeAg seroconversion) mark a transition from the immune-active phase of the disease to the inactive carrier state (Chen and Yang, 2011).

The liver secretes bile into the bile duct system, while a variety of gut-derived signals, such as bacterial products, environmental toxins, and dietary antigens, enter the gut and travel through the biliary tract to the liver (Albillos et al., 2020). Based on the “gut-liver axis, “an increasing number of research have discovered a link between hepatic damage and disturbance of gut microbial homeostasis. Growing data suggests that gut microbe-derived metabolites such as trimethylamine, bile acids, and short-chain fatty acids play a substantial role in the beginning and progression of liver diseases (Zhao et al., 2020; Jia et al., 2018). Furthermore, a large signaling network regulated by microbiota-derived metabolism continues to be studied, providing gut-liver targets for disease therapy (Li et al., 2023).

Intestinal microbiota transplantation (IMT) is a novel approach to restoring and reconstructing the intestinal microecological balance and diversity. IMT has been shown to be effective in the treatment of a variety of disorders, including *Clostridium difficile* infection, metabolic diseases, malignancies, autoimmune diseases, nonalcoholic fatty liver disease (NAFLD), and hepatic encephalopathy (Surawicz et al., 2013; de Groot et al., 2017; Vaughn et al., 2019; Meighani et al., 2020). Besides, IMT appears to be safe and potentially effective in terms of viral suppression and HBeAg clearance in patients with HBeAg-positive CHB (Chauhan et al., 2021).

Although some studies have been conducted on the diversity of gut microbiota in chronic hepatitis B, as well as the clinical effects of IMT on E antigen and liver cirrhosis, research on the effects of IMT on early stage cirrhosis or pre-cirrhosis patients and changes in the microbiota post-IMT is quite limited. Therefore, in the present study, we performed a clinical study to characterize gut microbiota in CHB progression, followed by an investigation into the role of IMT in HBeAg-negative CHB patients, and finally, we examined the impact of IMT on gut microbiota composition and metabolomics signature.

## Methods

### Subjects and study design

There were three study groups, a cross-sectional study was conducted to see if there was any alternation in gut microbiota and metabonomics in HBV-CLD patients with healthy controls. Afterward, a perspective study was performed in the cohort of patients before IMT and after IMT. The study was approved by The Research Ethics Committee at The Fifth Affiliated Hospital of Sun Yat-Sen University (no.·ZDWY [2023] Lunzi no.·K254-1). Written informed consent was obtained from each participant before the initiation of study procedures.

A total of 16 HBeAg-negative HBV-CLD patients were enrolled at The Fifth Affiliated Hospital of Sun Yat-Sen University from February 2022 to March 2024, including 3 early stage of cirrhosis and 13 pre-cirrhosis. HBV-CLD patients on oral antivirals, which included entecavir (ETV), tenofovir disoproxil fumarate (TDF) or tenofovir alafenamide fumarate (TAF) alone, for more than a year undetectable of serum levels of HBVDNA and those who consented for the IMT were included in the IMT arm. A total of 10 age-and BMI-matched Chinese healthy individuals were enrolled, The inclusion criteria were as follows: (a) alcohol free history; (b) smooth and soft stool that was sausage or snake shaped, and (c) voluntary participation in this study. Patients with co-infections with hepatitis C virus (HCV) or human immunodefciency virus (HIV), chronic kidney disease, hepatocellular carcinoma, underlying other comorbidities, and those unwilling to provide written informed consent for the IMT procedure were excluded. We also excluded patients with underlying cirrhosis.

Fecal microbiota capsules for the IMT group were obtained from healthy undergraduate donors (Guangdong Pharmaceutical University) and evaluated using comprehensive physical examinations. The preparation and the transplantation of fecal microbiota capsules were performed as previously described (Kao et al., 2017). Forty capsules were manufactured from 1 donation. All HBV-CLD patients received a total of 120 capsules, 6–10 capsules three times a day (according to the digestive reaction of patients, each dosage can be appropriately reduced), for a total course of about 6 days. Theoretically, there were four groups: Group A, HBV-CLD patients; group B, healthy controls; group C, pri-IMT; group D, post-IMT.

### Clinical data collection

All patients who underwent IMT were evaluated clinically daily while taking the fecal microbiota capsules for the development of any new symptoms, assessment of side-effects post-procedure. Adverse events in the form of eight gastrointestinal symptom parameters were observed within 7 days following IMT. Liver function tests, blood routine examinations, HBsAg, and HBVDNA levels were measured at baseline and weeks 8, 24. All 16 patients completed blood tests, 13 patients completed pri-IMT stool 16S rRNA sequencing, and 7 patients completed post-IMT 16S rRNA sequencing.

### 16S rRNA sequencing and bioinformatic analysis

Stool samples were collected before IMT and 1 month after IMT. Study participants were given a stool sample kit at each in-person visit to collect the stool sample. Explicit instructions were given to the participants on how to collect the samples. Once collected (within seven days of an inperson visit), the samples were frozen at −80°C until sequencing. Libraries were generated by amplification of the V3–V4 region of the 16S ribosomal RNA gene of DNA extracted from gut mucosal biopsies and from stool samples, and sequenced on the Illumina MiSeq platform (Illumina, San Diego, California). For 16S analysis, QIIME (version 1.9.1) was used to demultiplex and quality-filter the raw FASTQ files. Next, operational taxonomic units (OTUs) were generated and clustered using a 97% threshold and Usearch (version 7.1). After aligning with the gold database (v20110519), UCHIME (4.2.40) was used to filter out chimaeric sequences, and use arch_global was used to quantify the OTU abundances for each sample. For taxonomic analysis of each representative OTU, the Greengenes database (v201305) was used based on the RDP classifier (Version 2.2) with a 0.8 confidence value. OTUs were then assigned to different hierarchical levels and taxonomic relative abundance profiles were summarized.

### Metabolomic profiling

To understand the roles and interactions of proteins, metabolites, and genes in biological systems, the KEGG database was built. In this study, we used DIAMOND (v0.9.7) to annotate the representative sequences of gene sets with the KEGG database. To obtain the functional abundance at each level, we used the BLAST comparison parameters with an expectation of E < 1e−5. To find the metabolic pathway enrichment results for metabolites, enrichment analysis was carried out using their KEGG IDs. Pathway entries that were considerably enriched in significantly differently expressed metabolites relative to the background were identified using hypergeometric testing.

### Statistical analysis

To analyze differences in continuous variables, we employed the paired-samples t-test or the Wilcoxon signed-rank test. Pearson correlation analysis for gut microbiota post IMT, FDR for multiple correction. Microbial features (species and metabolites) found in fewer than 20% of the samples were eliminated prior to statistical analysis of the relative abundance data. Spearman’s rank correlation was used to examine associations between gut microbiota and metabolites, which were then adjusted for significance using the Benjamini-Hochberg approach. Linear discriminant analysis effect size (LEfSe) was used to assess groups for statistically significant species and functional differences. The nonparametric factorial Kruskal-Wallis test, Wilcoxon rank sum test, and LDA were used to identify biological and functional markers that were enriched for differences between multiple metadata categories. We performed statistical analyses using SPSS 22.0 (IBM Corporation, Armonk, New York, United States) and GraphPad Prism 6.0 (GraphPad Software, Inc. La Jolla, CA, United States). Unless otherwise stated, all statistical tests are two-tailed, and *p* < 0.05 was deemed statistically significant.

## Results

### Evaluation of clinical comprehensive curative effect of IMT on HBV-CLD patients

Between December 2023 and March 2024, 30 participants were screened and 26 were enrolled in this study: 16 HBV-CLD patients accepted IMT, 10 healthy controls. Two HBV-CLD patients were excluded for they did not complete the treatment course of IMT. Fourteen HBV-CLD patients received full course of IMT, while two patients did not complete blood tests.

**Figure 1:**
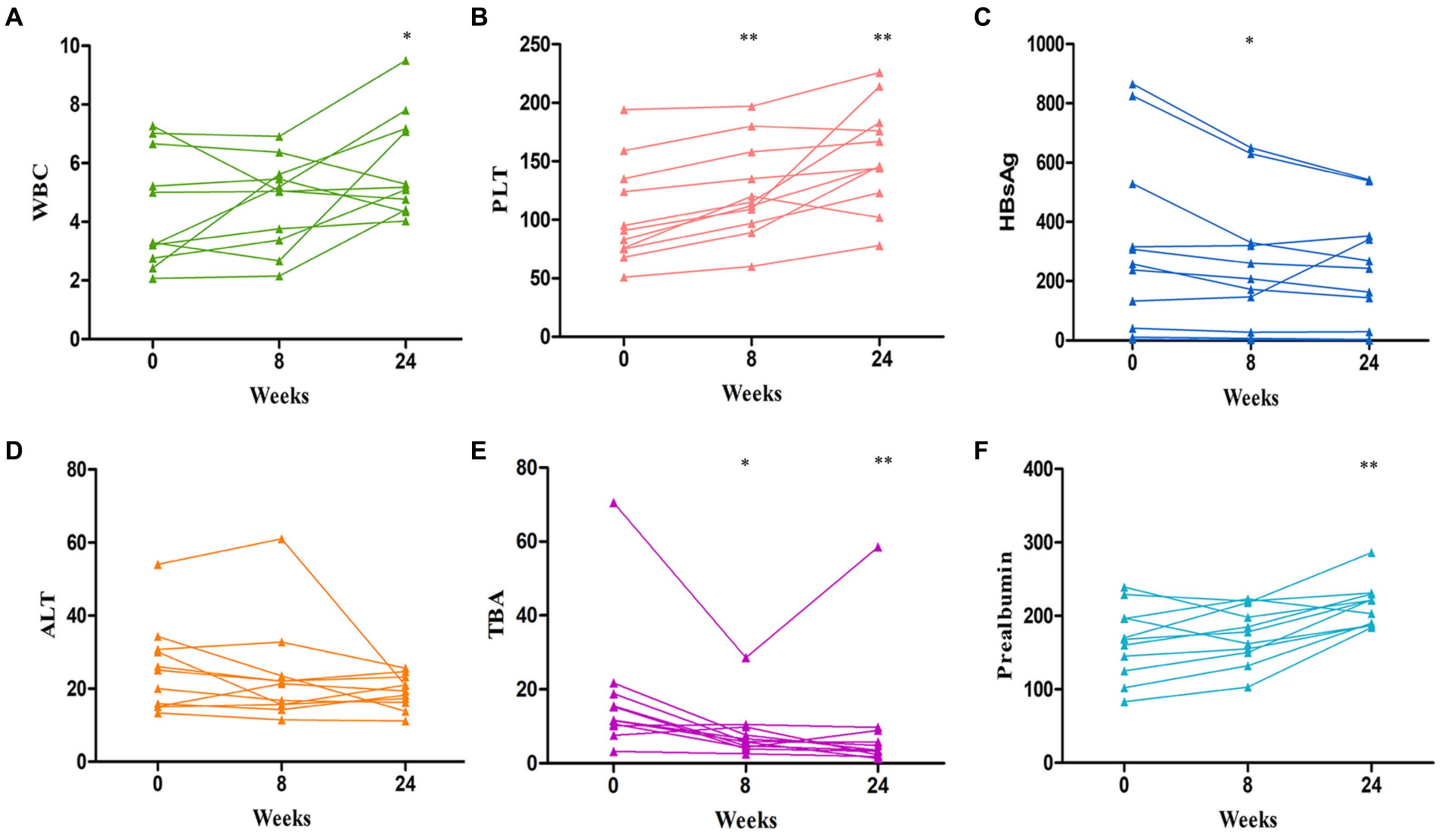
Changes curve in laboratory parameters over time post IMT. **(A)** the change curve of white blood cells (WBC); **(B)** the change curve of platelet (PLT); **(C)** the change curve of HBsAg; **(D)** the change curve of alanine aminotransferase (ALT); **(E)** the change curve of total bile acid (TBA); **(F)** the change curve of prealbumin. *p < 0.05, **p < 0.01.

All of the patients had undetectable HBVDNA at baseline and continued on their previous antiviral medication while undergoing IMT. 78.5% of patients had hypothrombocytemia, with a median of 75 × 10^9^/L. Plaque increased considerably after IMT at 8 weeks (*p* = 0.001) and 24 weeks (*p*<0.001) (Figure 1B). Additionally, 78.5% had hypoproalbuminemia, with a median of 145 g/L 8 weeks (*p* = 0.259), 24 weeks (*p* = 0.007) (Figure 1F), HBsAg showed a significant decrease after IMT: 8 weeks (*p* = 0.026), 24 weeks (*p* = 0.116) (Figure 1C), and total bile acid (TBA) showed a significant decrease after IMT: 8 weeks (*p* = 0.024), 24 weeks (*p* = 0.001) (Figure 1E). Some individuals with hypoleukocytoemia had an increase in white blood cells following transplantation (Figure 1A). Nonetheless, the alanine transaminase (ALT) (Table 1) and aspartate aminotransferase (AST) levels were mostly maintained in the normal range following IMT.

**Table 1:** Changes in laboratory parameters over time post IMT.

	Pri-IMT	Post-IMT 8 weeks	*p* value	Post-IMT 24 weeks	*p* value
WBC (×10^9/L)	4.3 ± 1.9	4.6 ± 1.5	0.495	5.8 ± 1.7	0.072
PLT (×10^9/L)	104.6 ± 43.4	116.9 ± 44.5	0.001	124.3 ± 42.7	<0.001
ALT (U/L)	25.3 ± 11.9	23.3 ± 13.7	0.307	19.2 ± 4.4	0.097
TBA (μmol/L)	17.8 ± 18.1	8.3 ± 7.2	0.024	9.4 ± 16.5	0.001
Prealbumin (mg/L)	164.9 ± 49.3	174.9 ± 38.7	0.259	214.7 ± 29.8	0.007
HBsAg (IU/ML)	320.7 ± 302.4	250.4 ± 224.5	0.026	238.8 ± 193.9	0.116

Data are presented as mean (SD). WBC, white blood cells; PLT, platelet; ALT, alanine aminotransferase; TBA, total bile acid.

### Alteration of gut microbiota composition in HBV-CLD patients pri-IMT and post-IMT

Alpha diversity indexes indicating community richness, diversity, evenness and coverage were assessed via Chaol, Shannon, Simpson, ACE and Coverage indexes. Therein, there was no difference in the Shannon index and Simpson index, but the Chao1 index was higher in the patient group. But, Chao1 places a strong emphasis on the presence of rare species within a community, with a primary focus on species richness or the total number of different species present. However, its drawback lies in the fact that it does not take into account the distribution abundance and evenness of species within the community. Both qualitative PCoA and PCA analysis were performed to evaluate β diversity. No significant changes in β diversity was observed in the comparison between HBV-CLD patients and healthy controls (Supplementary Figure S1).

**Figure 2:**
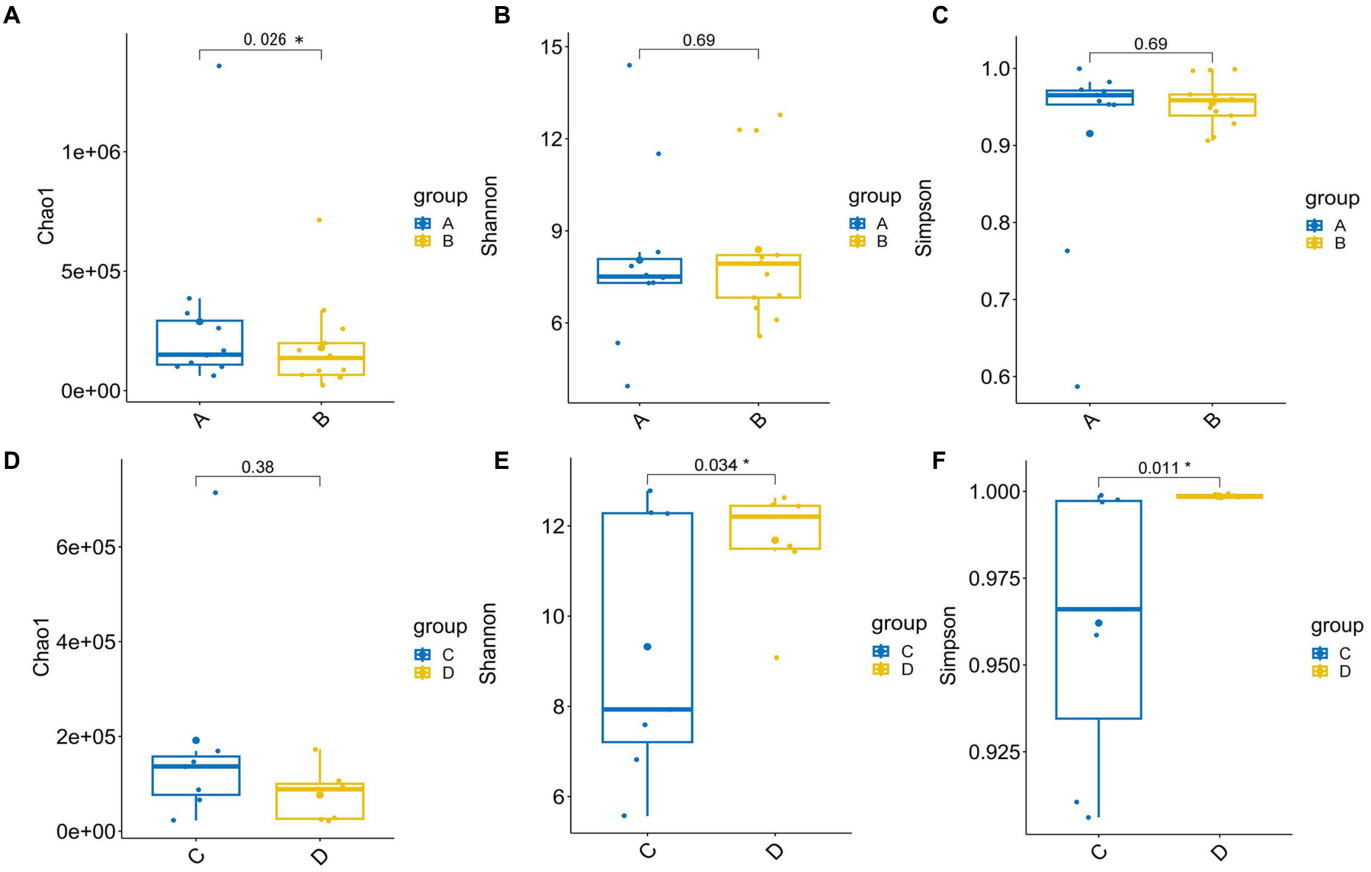
The α diversity of gut microbiota. The α diversity for HBV-CLD patients (group A) compared with healthy controls (group B) in Chaol indexes **(A)**, Shannon indexes **(B)**, Simpson indexes **(C)**, the α diversity for pri-IMT (group C) compared with post-IMT (group D) in Chaol indexes **(D)**, Shannon indexes **(E)**, Simpson indexes **(F)**. *p < 0.05.

There was significant difference in the Shannon and Simpson indexes between prior to IMT (pri-IMT) and post-IMT (Figures 2E,F; Shannon *p* = 0.034, Simpson *p* = 0.011), indicating that the impaired abundance of the gut microbiota had been improved after IMT in HBV-CLD patients. No significant changes in β diversity was observed in the comparison between pri-IMT and post-IMT (Supplementary Figure S2). In addition, we conducted pearson correlation analysis of individual microflora post-IMT. Although there was still a strong correlation between the microflora before and after transplantation, individual evolution occurred to different degrees (Table 2).

**Table 2:** Pearson correlation analysis of gut microbiota post IMT.

Number	Pri-IMT	Number	Post-IMT	Cor	*p* value
PRS2075200220	N1-pri IMT	PRS2075200475	N1-post IMT	0.78	0.00
PRS2075200473	N2-pri IMT	PRS2075200458	N2-post IMT	0.45	0.00
PRS2075200591	N3-pri IMT	PRS2075200433	N3-post IMT	0.8	0.00
PRS2075200470	N4-pri IMT	PRS2075200402	N4-post IMT	0.52	0.00
PRS2075200450	N5-pri IMT	PRS2075200404	N5-post IMT	0.66	0.00
PRS2075200454	N6-pri IMT	PRS2075200589	N6-post IMT	0.11	0.06
PRS2075200505	N7-pri IMT	PRS2075200482	N7-post IMT	0.54	0.00

Cor: Pearson correlation coefficient, FDR for multiple correction.

**Figure 3:**
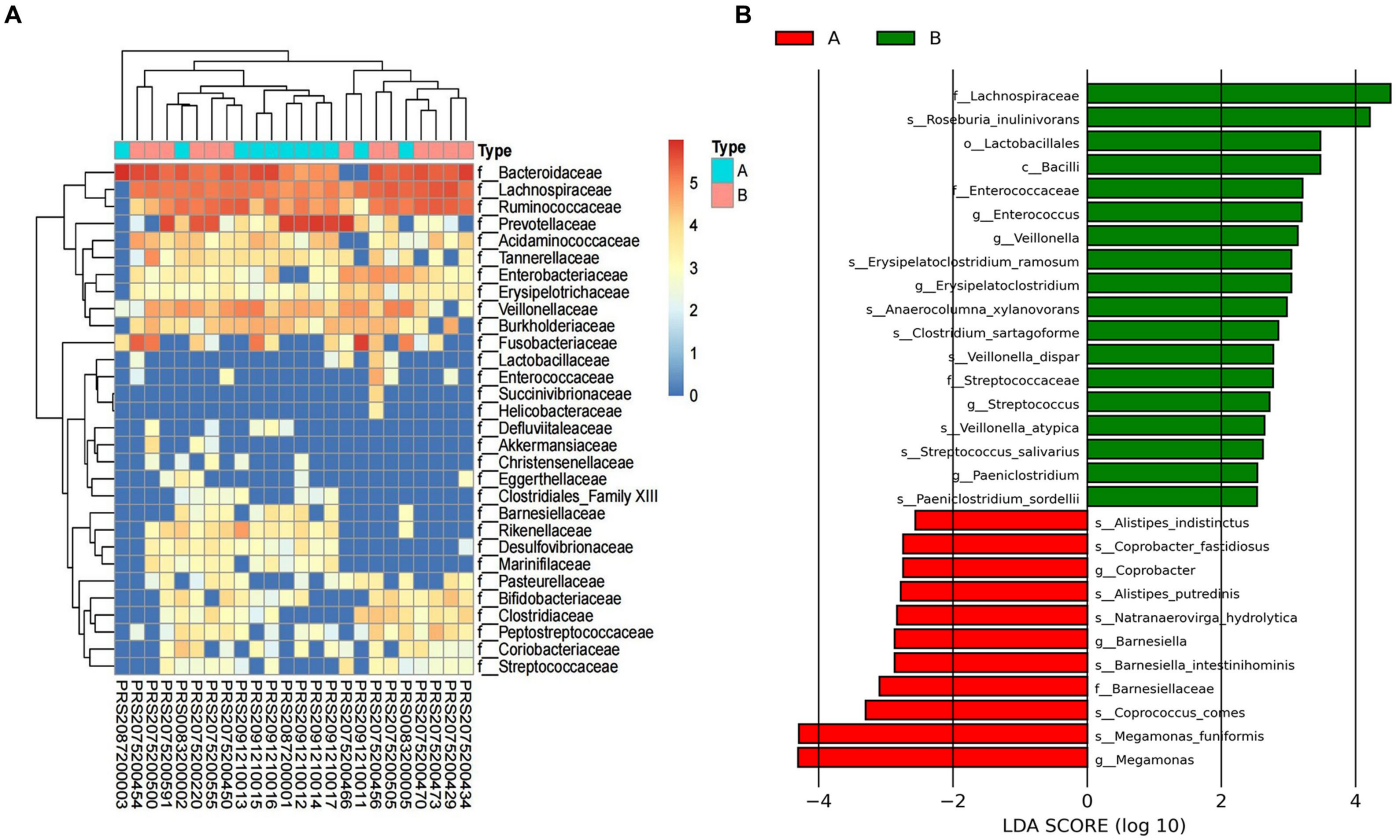
Gut microbiota profile for HBV-CLD patients. The relative abundance between group A and group B **(A)**. LEfSe analysis shows species with significant differences in abundance between group A and group B **(B)**. Group A: HBV-CLD patients, group B: healthy controls.

**Figure 4:**
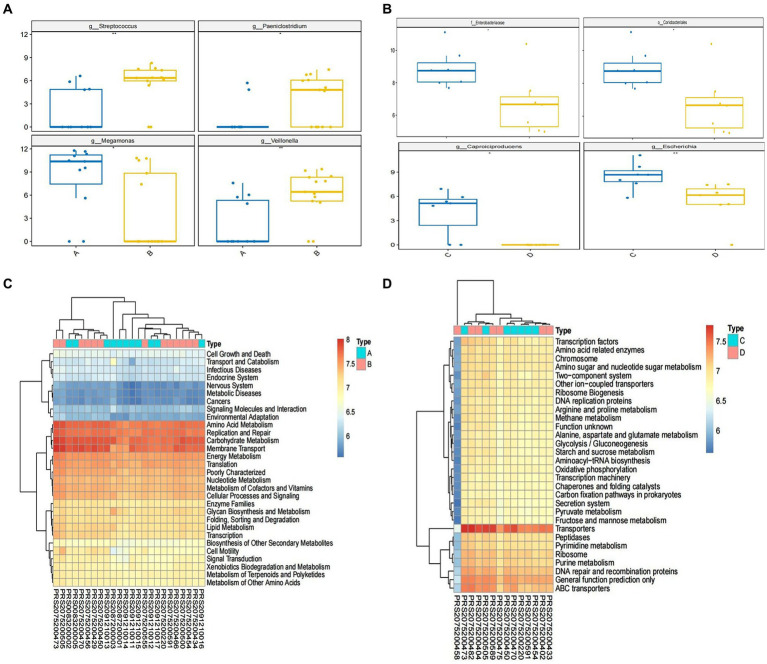
Alternation of microbiome and the predicted microbial functional composition with PICRUST analysis. The relative abundance of microbiome in genus level between group A and group B **(A)**; the relative abundance of microbiome in species level between group C and group D **(B)**. The predicted microbial functional composition with PICRUST analysis for HBV-CLD patients (level II) **(C)**. The KEGG pathways (level III) with PICRUST analysis after IMT **(D)**. Group A: HBV-CLD patients, group B: healthy controls, group C: pri-IMT, group D: post-IMT.

To further explore the taxonomic features and composition differences of gut microbiota associated with HBV-CLD patients, the raw macrogenome data were checked by quality control, filtered, and assembled to construct abundance profiles at the corresponding taxonomic levels. And we compared bacteria abundance before IMT and after IMT to further demonstrate the microbiota shifts in HBV-CLD patients. In LEfSe analysis (*p* < 0.05, q < 0.1, LDA > 2.0), compared with the HCs, the c. Bacilli, o. Lactobacillales, f. Streptococcaceae, g. *Streptococcus*, g. Paeniclostridium, g. Veillonella, were further diminished in CHB patients (Figure 3). While f. Rikenellaceae, f. Lachnospiraceae, f. Barnesiellaceae, g. Megamonas, g. Alistipes, g. Erysipelatoclostridium, were more enriched in CHB patients (Figures 4A,B).

**Figure 5:**
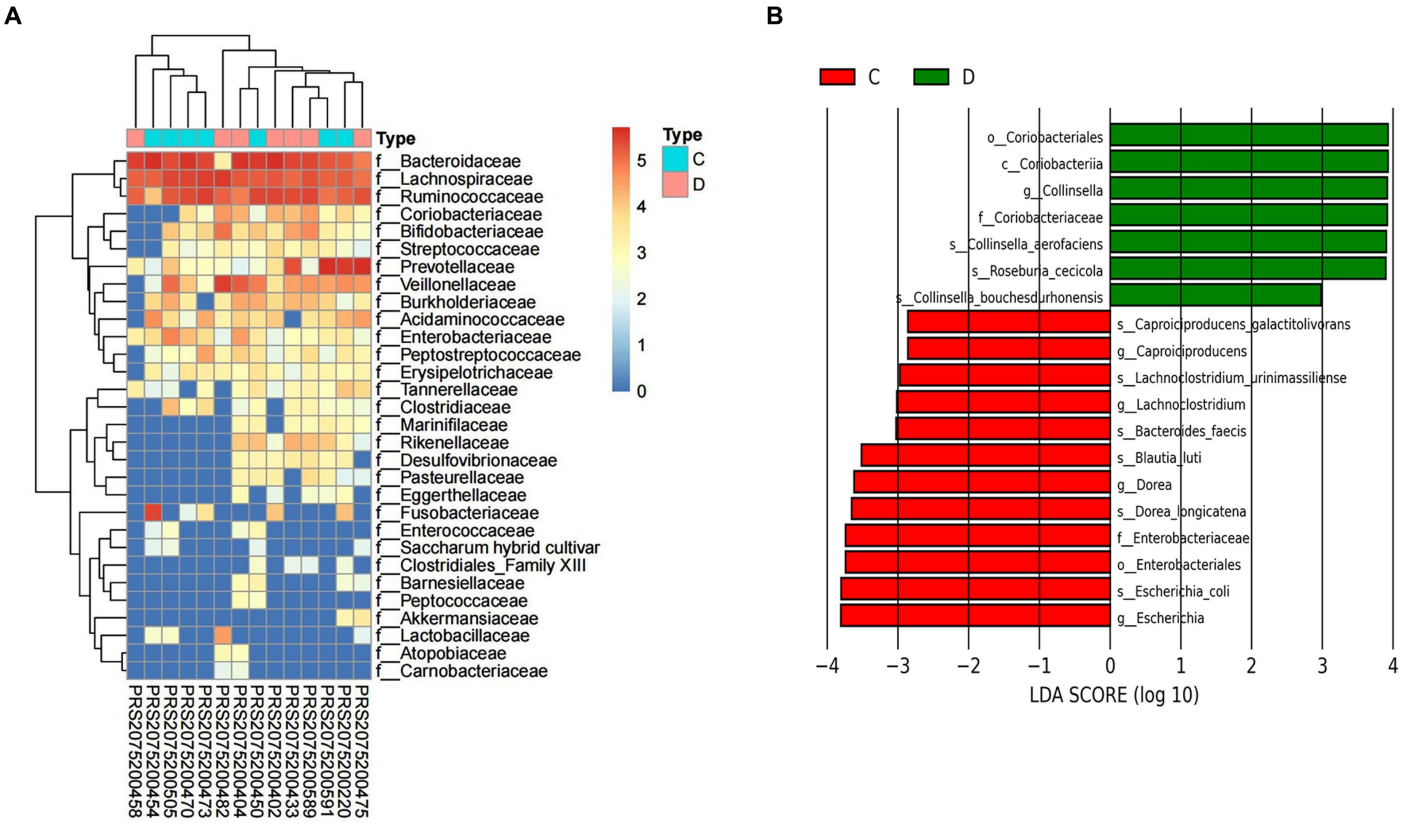
Alternation of gut microbiota composition after IMT. The relative abundance between group C and group D **(A)**. LEfSe analysis shows species with significant differences in abundance between group C and group D **(B)**. Group C: pri-IMT, group D: post-IMT.

After IMT, compared with pri-IMT, the o. Coriobacteriales f. Enterobacteriaceae, g. Collinsella, g. Lachnoclostridium, g. Caproiciproducens, were significantly decreased in post-IMT patients (Figure 5).

### Alteration of gut metabolites in HBV-CLD patients pri-IMT and post-IMT

To determine if the functional pathways involved in gut metabolites and microbiota are consistently changed, we ran pathway enrichment analysis on the KEGG IDs of metabolites and obtained pathway enrichment findings. Representatively various metabolites would be examined.

Compared to the HCs, amino acid metabolism, including alanine, aspartate, glutamate and tryptophan metabolism, was significantly decreased in CHB patients. In addition, the nicotinate and nicotinamide metabolism, starch and sucrose metabolism, steroid biosynthesis, and vitamins metabolism, were significantly lower in CHB patients than HCs. It is worth noting that oxidative phosphorylation and nucleotide excision repair were further depleted in CHB patients. Whereas p53 signaling pathway was more exuberantly in CHB patients than HCs (Figure 4C).

Following IMT, PICRUST analysis showed that the relative abundance of t the metabolites listed above were partially restored. In addition, the PPAR signaling pathway and pathogenic *Escherichia coli* infection significantly lower in post-IMT than pri-IMT (Figure 4D).

## Discussion

In this study, we investigated the effectiveness of IMT on HBV-CLD patients and characterized gut microbiota in HBV-CLD progression by combining microbiome with metabolome pri-IMT and post-IMT. HBV-CLD patients in our study presented with hypoproalbuminemia, hypersplenism such as leukopenia and thrombocytopenia, but no sequelae from decompensated cirrhosis. Long-term antiviral medication did not ameliorate these problems.

In this study, a decrease in HBsAg was observed in E antigen-negative patients post-IMT 8 weeks. There were reported that IMT appeared to be safe and potentially effective in terms of viral suppression and HBeAg clearance in patients with HBeAg-positive CHB, but achieved no HBsAg clearance (Chauhan et al., 2021; Ren et al., 2017). IMT has the potential to modulate the host’s immune response and alter its susceptibility to HBV infection (Wang et al., 2022).

Oral IMT capsules were safe and well-tolerated in patient with cirrhosis and recurrent HE; besides, gut microbial function in cirrhosis is beneficially affected by capsular FMT, with improved inflammation and cognition (Wang et al., 2022; Bajaj et al., 2019). For patients with HBV-CLD in this study, increase in platelets and white blood cells could be observed after 24 weeks. It has been proven that gut flora (including Bacteroidetes, Parabacteroides, and Proteobacteria) were restored to levels comparable to those reported in healthy persons following splenectomy, which was followed by an increase in WBC and PLT counts in liver cirrhosis patients (Liu et al., 2018). Furthermore, significant improvements in proalbumin could be observed post-IMT. PICRUST analysis revealed that IMT restored the relative abundance of KEGG pathways involved in amino acid metabolism. That might be contribute by the improved composition of the gut microbiota by IMT. Furthermore, IMT might contribute largely to slow the progress of liver impairment.

The α diversity of gut microbiota was significantly decreased in the HBV-CLD arm, but showed considerable rebuilding following IMT. The hepatic portal and bile secretion networks allow the gut to connect directly with the liver (Cesaro et al., 2011). Improved gut microbiota and metabolomics may help to minimize liver damage.

In this study, the HBV-CLD arm exhibited a decrease in families from the Lactobacillales and Bacillus orders. Bacteroides, Lactobacillus, and Clostridiumsedis were linked to survival in cirrhosis patients with hepatic encephalopathy during a one-year period (Sung et al., 2019). Lactobacillus GG and prebiotics/synbiotics could improve microbial composition and outcomes in patients with cirrhosis (Dhiman et al., 2014). Lactobacillus play a role in the conversion of primary bile acids through the production of bile salt hydrolase (Li et al., 2023).

In accordance with previous studies, the abundance of Clostridia was downregulated in HBV-CLD patients (Ren et al., 2019; Zeng et al., 2020). The main producers of butyrate are Clostridia, Eubacteria, and Roseburia microbes (Nicholson et al., 2012). Short-chain fatty acids (SCFAs) are saturated fatty acids containing one to six carbon atoms, including acetate, propionate and butyrate. SCFAs regulate liver immune homeostasis and lipid metabolism by activating G-protein-coupled receptors or inhibiting the activity of histone deacetylase (HDAC) (Koh et al., 2016). Butyrate plays an important role in the regulation of intestinal barrier function, intestinal immunity, and inflammation response. Butyrate can bind to and activate peroxisome proliferator-activated receptorγ (PPARγ), thereby exerting an antiinflammatory effect through the antagonism of nuclear factor-κB transcription (Cani et al., 2019). The recovery observed in the gut microenvironment following IMT reflects changes in not only the abundances of bacteria and metabolites but also in the correlations between them.

The abundance of Megamonas was up-regulated in HBV-CLD patients in this study. It has been reported that Macroomonas was more abundant in Asian populations with colorectal cancer (Yachida et al., 2019). In addition, the attention-deficit/hyperactivity disorder (ADHD) group showed higher levels of Dialister and Megamonas. IMT modified the abundance of the Megamonas. Notably, the abundance of *E. coli* was down-regulated, and PICRUST analysis showed that the pathogenic *Escherichia coli* infection significantly lower in post-IMT than pri-IMT. *E. coli* and *Streptococcus* are the main causes of bacterial infection in patients with cirrhosis (Riordan and Williams, 2010). Bacteria from Enterobacteriaceae family (including *Escherichia coli*, Klebsiella, Proteus) are all regarded as PAMPs-producing bacteria (Chen et al., 2011), and the intestinal microflora is the main source of portal LPS and represents an important prerequisite for the development of liver fibrosis in chronic liver injuries (Paik et al., 2003).

Several limitations should be acknowledged in our study. Firstly, the limited sample size of microbiome and metabolomics may restrict significance and stability of the results. A long-term follow-up of prospective cohorts is required to further validate potential taxa/metabolites. Secondly, it’s important to note that 16 s rRNA sequencing has limitations in providing gut microbiota annotations with accuracy at the ‘species’ level. Vague annotations may lead to contentious outcomes. Thus, short-gun sequencing of metagenomics and metatranscriptomics has the potential to disclose more accurate information about the microbial community’s makeup and function.

## Conclusion

In conclusion, HBV-CLD patients who underwent IMT showed improvements in liver function as well as a recovery of the composition and metabolism of the gut microbiota in this study. IMT results in decreased relative abundance of pathogenic bacteria such as Enterobacteriaceae and Megamonas. Furthermore, a increase in prealbumin and metabolomics of amino acid (including Eaa, tryptophan). The underlying mechanism still needs further study. Therefore, the regulation of gut microbiota by IMT may provide a new clinical approach for the treatment of HBV-CLD.

## Data Availability

The original contributions presented in the study are included in the article/Supplementary material, further inquiries can be directed to the corresponding authors.
