# Supplementary figures and images for: Effect of intestinal microbiota transplantation on chronic hepatitis B virus infection associated liver disease
Source: Front Microbiol. 2024 Sep 11;15:1458754. doi: 10.3389/fmicb.2024.1458754 (PMC11422146; doi:10.3389/fmicb.2024.1458754)

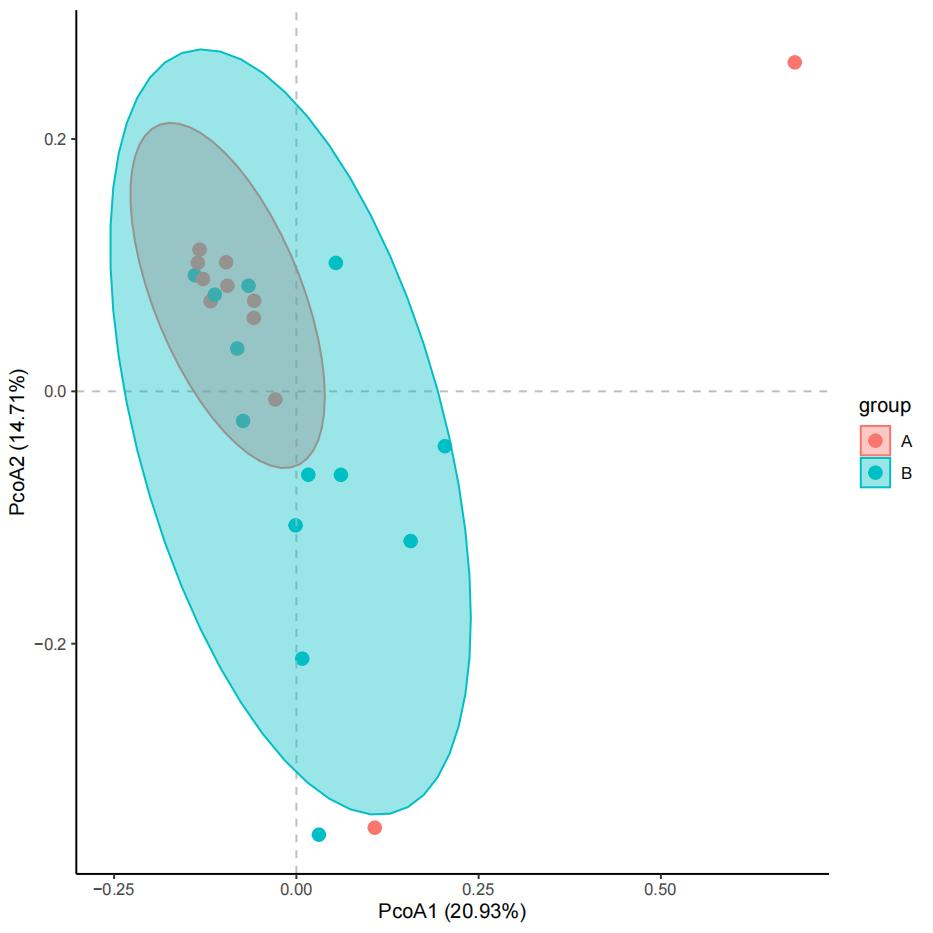

Supplement: Supplementary file 1 [file Image_1.JPEG]

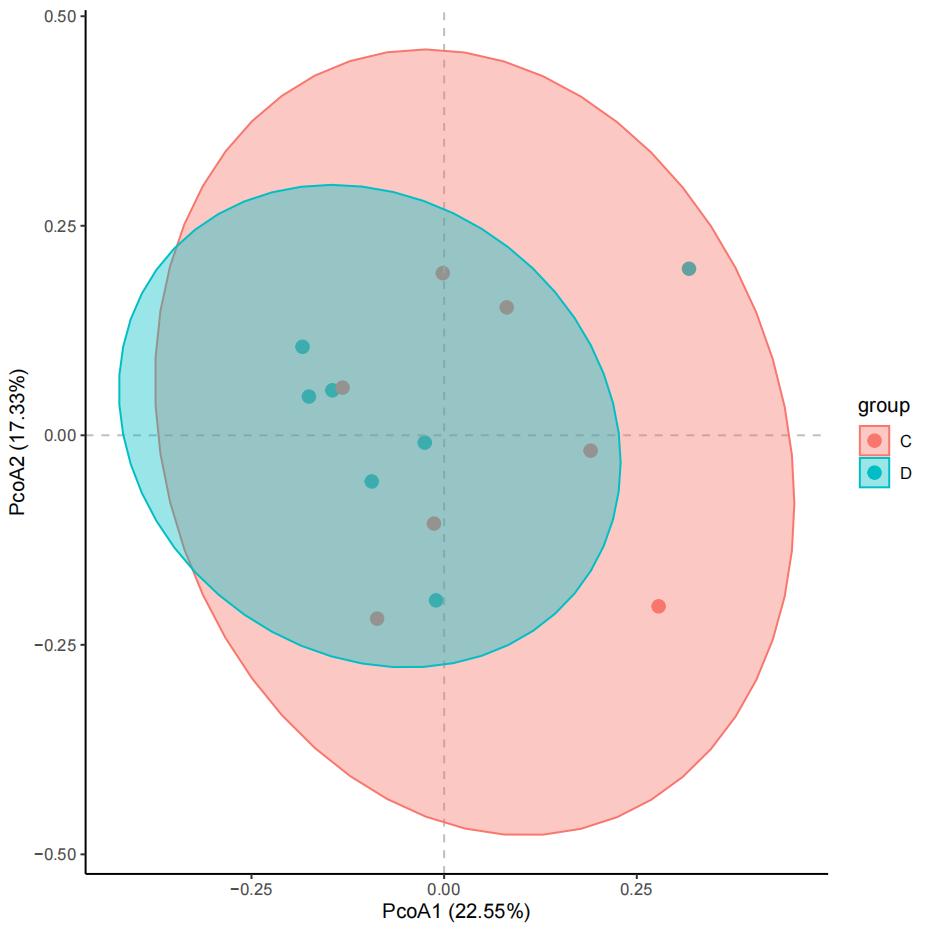

Supplement: Supplementary file 2 [file Image_2.JPEG]
